# Micronutrient Testing, Supplement Use, and Knowledge Gaps in a National Adult Population: Evidence from Saudi Arabia

**DOI:** 10.3390/nu17243897

**Published:** 2025-12-12

**Authors:** Abdulmajeed Fahad Alrefaei, Saeed M. Kabrah

**Affiliations:** 1 Department of Biology, Jamoum University College, Umm Al-Qura University, Makkah 2203, Saudi Arabia; 2 Department of Clinical Laboratory Sciences, Faculty of Applied Medical Sciences, Umm Al-Qura University, Makkah 2203, Saudi Arabia; smkabrah@uqu.edu.sa

**Keywords:** micronutrient deficiency, vitamin D, vitamin B12, iron, public health, Saudi Arabia

## Abstract

**Background:** Micronutrient deficiencies, particularly of vitamin D, vitamin B12, and iron, are prevalent worldwide and contribute significantly to morbidity. In Saudi Arabia, these deficiencies are increasingly recognised as public health challenges, yet comprehensive data on prevalence, risk factors, and public awareness remain limited. **Objectives:** This study aimed to determine the prevalence of laboratory-confirmed deficiency and testing for vitamin D, vitamin B12, and iron among adults in Saudi Arabia; to identify associated sociodemographic, lifestyle, and clinical factors; and to assess public knowledge and attitudes regarding micronutrient status. **Methods:** A cross-sectional survey was conducted in 2025 among adults residing in Saudi Arabia. Data were collected using a structured, self-administered questionnaire covering demographics, lifestyle, chronic disease history, laboratory testing and supplementation, and knowledge and perceptions regarding micronutrient deficiency. Descriptive statistics, chi-squared tests, and network analysis were used to identify associations and patterns. **Results:** A total of 1652 participants were included (52.6% female; mean age 41.3 ± 10.2 years). The prevalence of laboratory-confirmed deficiency was 7.6% for vitamin D, 5.5% for vitamin B12, and 7.0% for iron. Most participants had never been tested for these micronutrients. Vitamin D deficiency was significantly associated with gender, age, education, marital status, physical inactivity, and the presence of chronic conditions. Similar patterns were observed for vitamin B12 and iron. Public knowledge was primarily sourced from social media and internet sites; 38.1% of participants considered vitamin deficiency a public health concern, and 96.4% supported awareness campaigns. **Conclusions:** Micronutrient deficiencies remain common and under-recognised among adults in Saudi Arabia. There is a critical need to improve public awareness, expand routine laboratory testing, and develop targeted interventions to address identified risk groups and knowledge gaps. Our study is the first study to investigate the status of three key micronutrients (vitamin D, vitamin B12, and iron) in a single large sample. We also employed network to explore the complex factors associated with micronutrient deficiencies.

## 1. Introduction

Micronutrients are essential mediators of human physiology, contributing directly to body metabolism through their actions in growth, development, metabolic reactions, and immune functioning [1,2]. Micronutrient deficiencies, particularly vitamin D, vitamin B12, and iron, are prevalent public health concerns worldwide, including in Saudi Arabia. These deficiencies can lead to serious health complications, such as osteoporosis, anaemia, neurological disorders, and weakened immune function [3,4,5].

Micronutrient deficiencies are common causes of malnutrition globally, affecting more than 0.37 billion pre-school children and approximately 1 billion pregnant women, with the highest prevalence in Asia and Africa [6]. Moreover, it resulted in mortality in approximately 10% children [7]. Micronutrient inadequacies lead to the development of chronic diseases, impaired immunity, cognitive defects, and fatigue, resulting in a socioeconomic impact characterized by decreased educational achievements, reduced working capacity, and lower earning potential [7]. Vitamin D, B12, and iron are among the key micronutrients that regulate several physiological mechanisms [1].

Vitamin D, a secosteroid, is an important micronutrient in the form of a fat-soluble vitamin that comprises a B ring with a broken carbon bond at the 9th and 10th positions. It is further categorised as cholecalciferol (Vitamin D3) and ergocalciferol (Vitamin D2) due to the presence of a double bond between carbon 22 and 23, along with a methyl group on carbon 24 in Vitamin D2 [8]. The interaction of 1,25(OH)2D3 with nuclear vitamin D receptors and retinoic acid X receptor (RXR) modulates gene expression via vitamin D response element (VDRE) [9]. It plays a pivotal role in calcium and phosphate homeostasis by stimulating intestinal absorption of calcium via increased expression of calbindin, TRPV6, TRPM7, PMCA1b, and NCX1 transporters and suppresses parathyroid hormone effect, hence promoting bone mineralization [9]. It mediates its immune effect by increasing the expression of certain antimicrobial peptides, such as cathelicidin LL-37. It produces its anti-inflammatory effects by decreasing the expression of inflammatory cytokines (IL-6, IL-23, and TNF) [10]. It reduces the progression of atherosclerosis by regulating T-cell differentiation [11]. In cancer cells, it reduces proliferation by increasing the expression of CKIs, p21, and p2, which halts the transition of the cell cycle to the S-phase. It promotes apoptosis by suppressing the expression of the BCL-2 gene [12]. Its negative effect on renin levels regulates blood pressure, hence supporting its role in cardiovascular health [13].

Naturally, cholecalciferol is produced in skin from 7-dehydrocholesterol under the effect of ultraviolet B (UVB) radiation in epidermal keratinocytes, which is further hydroxylated via the liver and kidney to produce calcitriol (1,25-dihydroxyvitamin D3 or 1,25(OH)2D3). Additionally, milk, egg yolk, margarine, and fish liver oil are essential dietary sources of Vitamin D [14]. Although most of Vitamin D binds to Vitamin D binding protein (DBP), and only a small amount remains free in circulation, the megalin-cubilin receptor system mediates the cellular absorption of Vitamin D-DBP complex, especially in the renal system, where unnecessary wasting of vitamins from urine is reduced [15]. A deficiency of Vitamin D in children results in rickets, while in adults, it leads to osteomalacia, which can cause multiple fractures that require therapeutic replacement [16,17]. The adjunctive role of Vitamin D in various inflammatory diseases is also described in the literature [18].

Vitamin B12, an essential cofactor in human metabolism, is a water-soluble micronutrient comprising a tetrapyrrolic corrin ring with a central cobalt moiety. It includes cyanocobalamin and hydroxocobalamin, adenosyl cobalamin and methyl cobalamin [19]. In the cytoplasm, it mediates the conversion of homocysteine to methionine, hence reducing the cardiovascular effects and promoting methylation of DNA and RNA, formation of myelin, and synthesis of neurotransmitters by promoting the synthesis of S-adenosylmethionine (SAMe) [20]. In mitochondria, it regulates the conversion of methylmalonyl-CoA to succinyl-CoA in the Krebs cycle and influences lipid metabolism through epigenetic mechanisms [21]. It is known to inhibit endoplasmic reticulum stress pathway occurring after traumatic injury, stabilize microtubules, and initiate remyelination of nerve fibres [22]. Deficiency of Vitamin B12 leads to megaloblastic anaemia, and neurological symptoms like peripheral neuropathy, subacute combined spinal cord degeneration, and cognitive impairment. Certain animal-based foods like red meat, liver, milk, and egg are rich sources of vitamin B12 [23,24]. Therapeutically, it has been used in pernicious anaemia, atrophic gastritis, malabsorption syndrome, pancreatic insufficiency, and along with long-term use of metformin [25,26,27].

Iron, an essential micronutrient that exists as heme in the human body, comprises a central iron bound within protoporphyrin IX via four nitrogen atoms. It consists of two positions on iron, one binds with the amino acid residue, and the other binds to oxygen [28]. It mediates ATP production via the electron transport chain. Additionally, the iron-sulfur cluster is an essential component of aconitase in the TCA cycle, a key source of energy metabolism in humans [29]. It acts as a cofactor for the CYPP450 enzyme, which is involved in hormone synthesis and the metabolism of drugs [30]. Ribonucleotide reductase, a DNA synthesis and repair enzyme, is also iron-dependent for activation of its catalytic site [31]. It plays an essential role in the development of memory, cognition, and learning by NMDA Receptor-Mediated Calcium Signalling [32]. The primary role of Iron is serving as a critical component of haemoglobin, the protein responsible for oxygen transport in red blood cells [33]. Beyond oxygen delivery, iron is a cofactor for numerous enzymes involved in cellular respiration. It is also a component of myoglobin, which facilitates oxygen storage in muscle tissue [6]. Dietary sources of iron include heme (red meat and fish) and non-heme iron (plant-based foods such as leafy greens) [34].

Iron deficiency results in anaemia, poor development and cognition in children, and preterm births in pregnancy. It leads to neurodegeneration through decreased myelin synthesis, impaired synaptogenesis, and aberrant functioning of the basal ganglia. It affects learning, memory, and cognition by modulating neuronal ferroptosis [32]. For example, Malabsorption syndromes and specific dietary habits can also contribute to deficiency [6]. Iron deficiency anaemia, the most severe manifestation, is characterized by a reduction in red blood cell count and haemoglobin levels, leading to fatigue, weakness, pallor, shortness of breath, and cognitive impairment. In children, iron deficiency can impair cognitive and motor development, while in adults, it can increase susceptibility to infections and reduce work capacity. Severe and prolonged iron deficiency can result in cardiac complications [6].

Despite the high prevalence, awareness regarding micronutrient deficiencies, their causes, symptoms, and preventive measures remains unclear among the Saudi population, especially regarding Vitamin D, B12, and iron. This study, therefore, aimed to (1) assess the prevalence of vitamin D, vitamin B12, and iron testing and deficiency among adults in Saudi Arabia; (2) identify sociodemographic, lifestyle, and clinical correlates of micronutrient status; and (3) evaluate public knowledge, attitudes, and support for awareness campaigns concerning micronutrient deficiency. The findings of this study will lead to the understanding of the awareness levels of vitamin D, B12, and iron deficiencies among the Saudi population.

This study is significantly important because it provides essential, evidence-based data on the prevalence of three major micronutrient deficiencies (vitamin D, B12, and iron) among the adult population in the kingdom of Saudi Arabia. It establishes an essential baseline where detailed information is limited. Identifying specific sociodemographic and lifestyle factors, these findings enable health authorities to move from general awareness to planning targeted strategies for the at-risk groups. Further, our findings highlight a significant public health concern because of the large numbers of untested individuals and validating a strong public demand for awareness campaigns. Finally, this research provides the necessary data to update the standard laboratory testing practices and help formulate evidence-based national strategies for prevention and improved nutritional health in the Saudi population.

In our study, the integration of deficiency rates, testing prevalence, knowledge assessment, and network analysis within a single, large sample is a key strength. This approach allows us to emphasize critical, novel public health insight: the persistent gap between public knowledge and actual micronutrient testing behavior.

## 2. Methods

### 2.1. Ethical Considerations

The study protocol was reviewed and approved by the Institutional Review Board of Umm Al-Qura University (Approval No: HAPO-02-K-012-2025-03-2621). Participation was voluntary, and electronic informed consent was obtained from all respondents before survey completion. Data were anonymised to ensure participant confidentiality and were used solely for research purposes.

### 2.2. Study Design and Setting

A cross-sectional survey was conducted to evaluate the prevalence of micronutrient testing and deficiency, associated socio-demographic and clinical factors, sources of knowledge, and public attitudes towards vitamin deficiency among adults residing in Saudi Arabia (Supplementary Materials File S1). Data collection was conducted between April 2025 and August 2025.

### 2.3. Study Population and Sampling

The required sample size for this study was estimated using an online sample size calculator (Calculator.net). Assuming a 95% confidence level, a 5% margin of error, a population proportion of 50% (to maximise sample size in the absence of prior prevalence data), and an estimated adult population of 32 million in Saudi Arabia, the minimum number of participants needed was calculated to be 385. The final analytic sample consisted of 1652 respondents, thereby substantially exceeding the minimum requirement and enhancing the precision and reliability of the study’s findings. The selected population comprised adults aged 18 years and above, residing in various regions and cities across Saudi Arabia. Participants were recruited using a combination of convenience and snowball sampling, with survey links distributed electronically via social media platforms (e.g., WhatsApp, Twitter), email lists, and institutional networks. The inclusion criteria included being at least 18 years of age, residing in Saudi Arabia, and providing informed consent.

### 2.4. Data Collection

A structured, self-administered questionnaire was developed and distributed in Arabic. The instrument was informed by previous surveys and literature on micronutrient awareness and deficiency in the Saudi Arabian population context. The questionnaire comprised five sections: Demographic and Socioeconomic Data, including, gender, age, marital status, education level, and city of residence. Lifestyle and Health Information: Exercise frequency, smoking status, and dietary habits (primary food source, vegetarian, high protein, and fast food diet). Clinical History: Self-reported presence or absence of chronic conditions, including hypertension, diabetes, anaemia, osteoporosis, heart disease, and high cholesterol. Micronutrient Testing and Supplement Use: Self-reported history of laboratory testing for vitamin D, vitamin B12, and iron, with response categories: “Below normal”, “Normal”, “Not tested”, and “Don’t know” (for iron only). Use of vitamin and mineral supplements was also assessed. Knowledge and Attitudes: Sources of knowledge regarding vitamins and micronutrients, perceived public health importance of vitamin deficiency, and support for awareness campaigns.

The face and content validity of the questionnaire were further established through review by a panel of five professionals with expertise in clinical nutrition, epidemiology, and public health. Each expert independently assessed the instrument for clarity, relevance, and comprehensiveness in addressing the study objectives. Feedback from the expert panel resulted in minor modifications to item phrasing and content, ensuring that the questionnaire was both contextually appropriate and scientifically robust. The questionnaire underwent pilot testing among 30 participants to assess clarity, comprehension, and timing. Minor revisions were made in response to feedback.

### 2.5. Data Management and Variable Definition

Survey responses were exported to Microsoft Excel and then imported into Python (version 3.10) for analysis. Age was categorised into five groups (18–25, 25–35, 35–45, 45–55, >55 years). Education level was categorised into four groups: less than high school, high school, university, and postgraduate. City of residence was recorded as a categorical variable, and responses were coded accordingly. Lifestyle factors (exercise, diet, and smoking status), chronic disease history, and supplement use were analysed as binary or categorical variables. For micronutrient testing, responses were classified as “Below normal” (deficiency), “Normal”, or “Not tested”.

### 2.6. Statistical Analysis

Descriptive statistics were used to summarise participant characteristics, prevalence of micronutrient testing, and deficiency rates. Frequencies and percentages were calculated for categorical variables. Associations between participant characteristics and micronutrient status (vitamin D, vitamin B12, iron) were evaluated using the chi-squared (χ^2^) test. Where appropriate, *p*-values less than 0.05 were considered statistically significant. For associations involving binary or categorical variables, Cramer’s V was computed to estimate the strength of association.

Correlation matrices and network graphs were constructed to visualise the relationships between key variables, with a threshold of Cramer’s V > 0.3 used to indicate strong associations. Bar plots were generated to illustrate the strength of association between individual features and micronutrient status. All analyses were conducted using Python (pandas, numpy, scipy, matplotlib, seaborn, and networkx libraries).

## 3. Results

### 3.1. Sociodemographic Characteristics of the Study Population

A total of 1652 individuals participated in the study (Table 1). The age distribution of the sample was broad, with the majority of respondents aged between 35 and 45 years (32.4%), followed by those in the 45- to 55-year age group (25.1%) and the 25- to 35-year age group (21.7%). Participants aged 18 to 25 years accounted for 12.9% of the sample, and those aged 55 and older comprised 7.9%. Regarding gender, the sample was nearly balanced, comprising 52.6% females and 47.4% males. The vast majority of participants were married (82.3%), while 12.8% reported being single and 5.0% fell into the ‘other’ category. In terms of educational attainment, 73.7% of participants had completed university-level education, 16.0% held a postgraduate qualification, and 10.2% had completed high school. Only a very small minority (0.1%) reported less than a high school education. Participants were geographically diverse, representing a wide range of provinces across the Kingdom of Saudi Arabia (Table 1 and Table S1). The most significant proportion of respondents resided in Makkah (21.5%) and Eastern Provinces (19.8%), followed by Al-Jawf Province (12.1%), Asir (9.6%), Al-Qassim (7.1%), and Tabuk (5.7%). Other notable provinces included Riyadh (5.4%), Al-Madinah (4.1%), Jazan (3.9%), Hail (3.8%), and Najran (3.3%).

### 3.2. Prevalence of Micronutrient Deficiency and Testing

Testing for vitamin D, vitamin B12, and iron was not routinely performed among the study population (Table 2). For each of the three micronutrients, the majority of participants reported that they had never undergone laboratory testing. For vitamin D, only 30.8% of respondents reported having ever been tested. Among these, 23.2% had normal results, whereas 7.6% were found to have results below the normal reference range. Nearly seven in ten participants (69.2%) had never been tested for vitamin D deficiency. Similarly, 31.1% of participants had undergone vitamin B12 testing, with 25.6% having normal results and 5.5% reporting results below normal. The majority (68.9%) reported never having been tested for vitamin B12 status. Iron testing was marginally more frequent, with 35.1% of the population reporting having had an iron test. Among those tested, 28.1% had normal results, 7.0% had results below normal, and 0.1% were unsure of their result. Nevertheless, 64.8% of the study population had never been tested for iron status. Interestingly, in our sample, 44.2% of participants exercised less than 3 times in a week (Figure S1).

### 3.3. Associations Between Participant Characteristics and Micronutrient Status

Significant variation in vitamin D status was observed across age groups (χ^2^ = 21.17, *p* = 0.020) (Table 3a). The prevalence of below-normal vitamin D levels was highest among participants aged 35–45 years (2.8%) and lowest among those aged 65 years and older (0.1%). Gender was also significantly associated with vitamin D status (χ^2^ = 8.35, *p* = 0.015), with females exhibiting a higher proportion of below-normal results (4.3%) compared to males (3.3%). Marital status was strongly associated with vitamin D status (χ^2^ = 43.64, *p* < 0.001), with married participants exhibiting a higher proportion of normal results (19.9%) compared to single or other groups. Educational level demonstrated a significant association (χ^2^ = 16.29, *p* = 0.012); participants with a university degree had the highest prevalence of both normal (16.0%) and below-normal (5.0%) vitamin D results. Lifestyle factors, including exercise frequency and smoking status, were also significantly linked to vitamin D status (χ^2^ = 44.37, *p* < 0.001 and χ^2^ = 9.11, *p* = 0.011, respectively). Participants who exercised 3–5 days per week were more likely to have normal vitamin D levels, whereas those who reported no exercise had a higher prevalence of vitamin D levels below normal. Dietary habits showed significant associations. Those consuming a vegetarian diet had a lower prevalence of below-normal vitamin D compared to non-vegetarians (0.8% vs. 6.8%, χ^2^ = 17.74, *p* < 0.001). A significant relationship was also noted between primary food source and vitamin D status (χ^2^ = 13.69, *p* = 0.008), with individuals mainly consuming home-cooked meals having a higher proportion of normal vitamin D results. Vitamin D status was significantly associated with the presence of chronic conditions such as anaemia (χ^2^ = 46.07, *p* < 0.001), osteoporosis (χ^2^ = 19.37, *p* < 0.001), high cholesterol (χ^2^ = 53.24, *p* < 0.001), other diseases (χ^2^ = 9.42, *p* = 0.009), and high blood pressure (χ^2^ = 20.70, *p* < 0.001). Participants with these conditions exhibited a higher frequency of results below the normal range for vitamin D.

The prevalence of below-normal vitamin B12 (Table 3b) was significantly associated with exercise frequency (χ^2^ = 24.12, *p* < 0.001), smoking status (χ^2^ = 6.07, *p* = 0.048), and high cholesterol (χ^2^ = 12.27, *p* = 0.002). Participants who engaged in more frequent exercise and those without high cholesterol exhibited a lower prevalence of deficiency. Notably, vitamin B12 deficiency was significantly more common among individuals with anaemia (χ^2^ = 11.42, *p* = 0.003) and those with high blood pressure (χ^2^ = 10.91, *p* = 0.004). Diabetes status also displayed a significant association (χ^2^ = 9.02, *p* = 0.011), with diabetic participants showing a higher proportion of below-normal vitamin B12 levels.

Iron deficiency (below-normal iron test results) was most common in those aged 35–45 years and showed no significant association with gender, education level, or most dietary habits (Table 3c). However, anaemia was strongly associated with below-normal iron levels (χ^2^ = 195.30, *p* < 0.001), with 2.1% of anaemic participants exhibiting iron deficiency compared to 5.0% of non-anaemic individuals. A significant association was also found between iron status and the presence of heart disease (χ^2^ = 30.52, *p* < 0.001), high cholesterol (χ^2^ = 46.68, *p* < 0.001), and high blood pressure (χ^2^ = 15.65, *p* = 0.001). Other diseases did not show significant relationships with iron status.

### 3.4. Knowledge Level Regarding Vitamin D, Vitamin B12, and Iron: Deficiency Symptoms, Sources, and Supplementation

**Figure 1:**
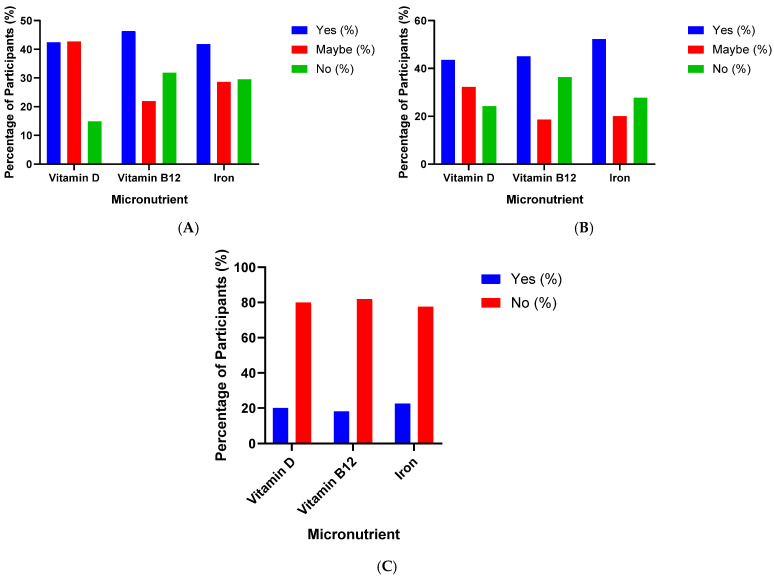
(**A**) Knowledge of deficiency symptoms, (**B**) Knowledge of food sources, and (**C**) Supplement intake of vitamin D, vitamin B12, and iron among participants (*n* = 1652). Bars represent the proportion of participants answering “Yes”, “Maybe”, or “No” to each question.

The study assessed participants’ knowledge regarding the symptoms, dietary sources, and supplementation practices for three essential micronutrients: vitamin D, vitamin B12, and iron (Table 4, Figure 1).

Regarding vitamin D, 42.4% of respondents correctly identified the symptoms of deficiency, whereas 42.7% were uncertain and 14.9% reported no knowledge. Similarly, 43.5% demonstrated awareness of food sources rich in vitamin D, 32.3% were unsure, and 24.2% did not know. Only one-fifth (20%) of participants reported taking vitamin D supplements. In addition, 54.2% of participants exposed to sunlight more than 30 min, while 41.6% of them chose less than 10 min of exposure time (Figure S3).

Knowledge of vitamin B_12_ was slightly higher: 46.3% recognised deficiency symptoms, 31.8% were uncertain, and 21.9% lacked awareness. In terms of dietary sources, 45% of respondents correctly identified vitamin B12-rich foods, 18.6% were unsure, and 36.4% did not know. Supplement use was reported by 18.1% of participants.

For iron, 41.8% of respondents were aware of deficiency symptoms, 28.6% were uncertain, and 29.5% reported no knowledge. Over half of the participants (52.2%) could identify iron-rich dietary sources, 20% were unsure, and 27.7% did not know. Iron supplementation was reported by 22.5% of participants.

Overall, the results indicate a moderate level of public awareness regarding micronutrient deficiencies, with knowledge of vitamin B12 being marginally higher than that of vitamin D and iron. However, the relatively low rates of supplement use highlight a gap between knowledge and preventive health behavior. One of important result is that 70.2% of participants chose mix diets (vegetarian diet and high protein diet) (Figure S2).

### 3.5. Associations Between Clinical, Behavioral, and Sociodemographic Variables and Laboratory Findings

**Figure 2:**
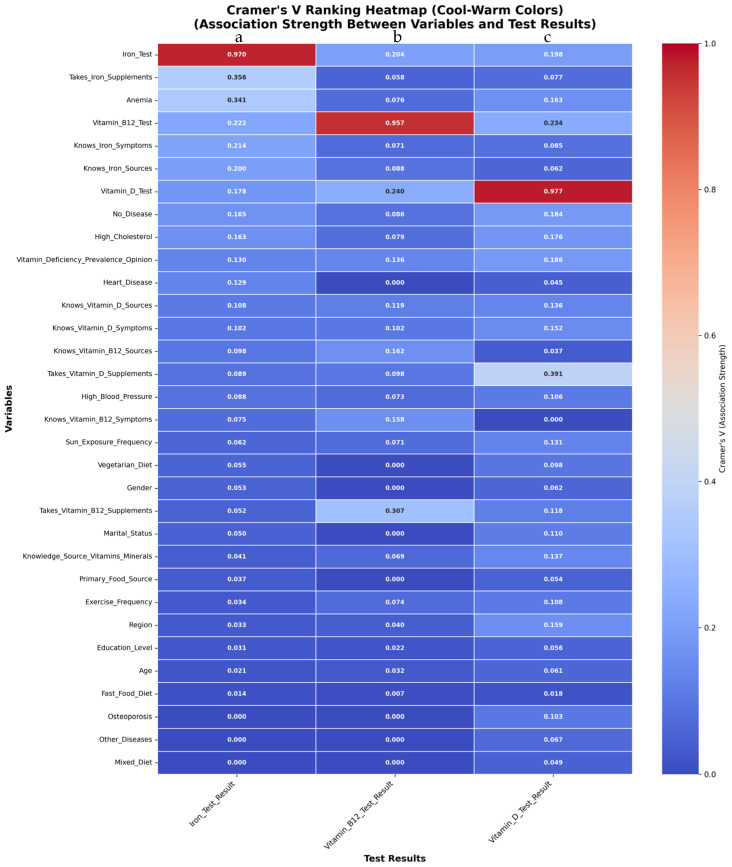
Combined Cramér’s V heatmap showing associations between participant characteristics and micronutrient test results. Each cell represents the Cramér’s V value for the association between a given variable and a specific laboratory test result. Darker shades indicate weaker associations, whereas lighter shades (towards warm colors) represent stronger relationships. Values range from 0 (no association) to 1 (perfect association). (**a**) Associations between all variables and iron test results; (**b**) associations between all variables and vitamin B12 test results; (**c**) associations between all variables and vitamin D test results. This figure enables comparison of the relative contribution of demographic, lifestyle, disease, behavioural, and knowledge-related factors to each micronutrient outcome.

A comprehensive analysis was undertaken to evaluate the strength of association between selected sociodemographic, clinical, dietary, behavioral, and knowledge-based variables and the results of laboratory assessments for iron, vitamin B12, and vitamin D (Figure 2). Associations were quantified using Cramér’s V, allowing for the identification of the most strongly related factors for each laboratory outcome.

Among all candidate predictors, the results of the self-reported iron test demonstrated the strongest associations with a select number of variables (Figure 2a). The highest Cramér’s V coefficient was observed between ‘Iron_Test’ and ‘Iron_Test_Result’ (Cramér’s V = 0.970), reflecting the expected concordance between the test and its result. Substantial associations were also evident for ‘Takes_Iron_Supplements’ (Cramér’s V = 0.356) and ‘Anemia’ (Cramér’s V = 0.341), indicating a close relationship between self-reported iron supplementation, clinical history of anemia, and laboratory-confirmed iron status. Additional variables with moderate strength of association included knowledge of iron deficiency symptoms and sources, results of vitamin B12 testing, and the presence of other chronic conditions. In contrast, variables such as education level, age, fast food consumption, and support for awareness campaigns exhibited only negligible associations with iron test results.

Analysis of predictors of vitamin B12 deficiency (Figure 2b) revealed that the most prominent association was between ‘Vitamin_B12_Test’ and ‘Vitamin_B12_Test_Result’ (Cramér’s V = 0.957), again reflecting the inherent dependency of the result upon the test. ‘Takes_Vitamin_B12_Supplements’ (Cramér’s V = 0.307) and ‘Vitamin_D_Test’ (Cramér’s V = 0.240) also exhibited relatively strong associations, suggesting potential overlap in the behavioral or clinical profiles of participants who receive these tests or supplements. Other moderately associated features included knowledge and awareness of vitamin B12 and vitamin D, as well as various dietary and health status indicators. Sociodemographic variables such as gender, marital status, and age contributed minimally to the variance in vitamin B12 status (Figure 2b).

When examining vitamin D status (Figure 2c), the most significant association was reported between ‘Vitamin_D_Test’ and ‘Vitamin_D_Test_Result’ (Cramér’s V = 0.977), consistent with expectations. ‘Takes_Vitamin_D_Supplements’ (Cramér’s V = 0.51) and ‘Vitamin_B12_Test’ (Cramér’s V = 0.391) were also among the more strongly related variables, suggesting a possible clustering of health behaviors or testing practices within the population. Knowledge variables and dietary factors, including sun exposure and consumption patterns, displayed moderate associations. Sociodemographic variables such as gender and age again had only a minimal relationship with vitamin D laboratory results (Figure 2c).

Overall, these findings demonstrate that self-reported supplementation, clinical history, and specific knowledge domains are the primary features associated with laboratory-confirmed micronutrient status, whereas sociodemographic variables play a much lesser role.

### 3.6. Network Analysis of Strong Categorical Associations

**Figure 3:**
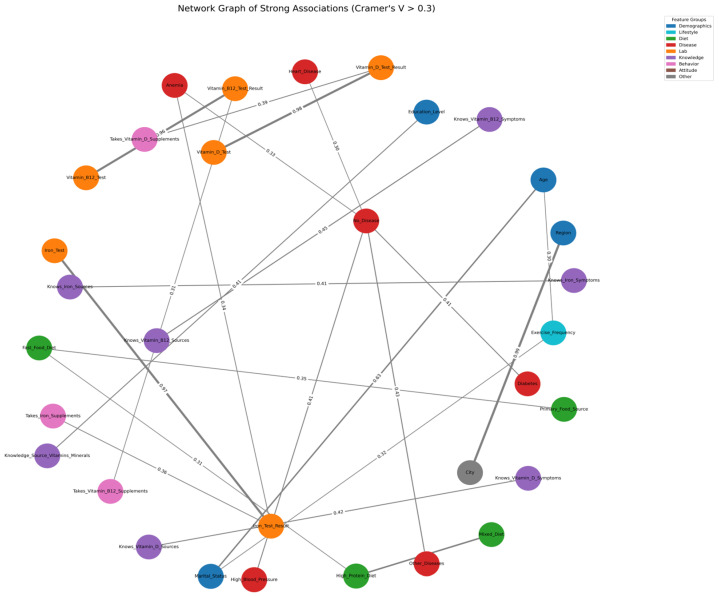
Network Graph of Strong Categorical Associations (Cramér’s V > 0.3). Nodes represent study variables, colour-coded by feature group: Demographics (blue), Lifestyle (cyan), Diet (green), Disease (red), Laboratory (orange), Knowledge (purple), and Behaviour (pink). Edges correspond to pairwise associations with Cramér’s V exceeding 0.3; edge width is proportional to the strength of association. Numeric labels on the edges indicate Cramér’s V coefficient for each pairwise association.

The network analysis (Figure 3) revealed several coherent clusters, reflecting the relationships between different domains of variables. Laboratory indicators formed the central and most prominent cluster, with each micronutrient test strongly linked to its corresponding test result. The iron, vitamin B12, and vitamin D clusters were interconnected with their respective supplementation behaviors, illustrating that individuals tested for a micronutrient were more likely to report taking related supplements. These laboratory–behavior links also extended to relevant knowledge variables, such as awareness of micronutrient sources and symptoms of deficiency.

A second cluster was formed by clinical disease variables, particularly anaemia, diabetes, high blood pressure, and self-reported absence of disease (“No_Disease”). These variables demonstrated moderate associations both within the disease group and with certain micronutrient-related features, underscoring the clinical relevance of micronutrient status in individuals with chronic conditions. Knowledge-related nodes attached to this cluster suggested that chronic illness may influence awareness of micronutrient health.

Dietary patterns formed a distinct cluster, with mixed diet, high-protein diet, and fast-food intake showing internal connectivity. These variables were moderately associated with education level and lifestyle behaviors, indicating patterned nutritional habits within the population.

Within the demographic domain, age and marital status were closely paired, forming a small but distinct cluster. The region also demonstrated moderate links to vitamin B12 testing behavior, hinting at geographic variation in health-seeking practices. Lifestyle variables such as exercise frequency occupied a peripheral but connected position, linking demographic factors with vitamin D testing and diet.

Overall, the network demonstrated a modular structure in which micronutrient testing and supplementation behaviors formed the core, surrounded by clusters representing chronic disease, diet, demographic patterns, and knowledge. This organisation highlights the interdependence of health behaviors, clinical status, and awareness within the study population.

### 3.7. Sources of Knowledge and Public Perceptions

**Figure 4:**
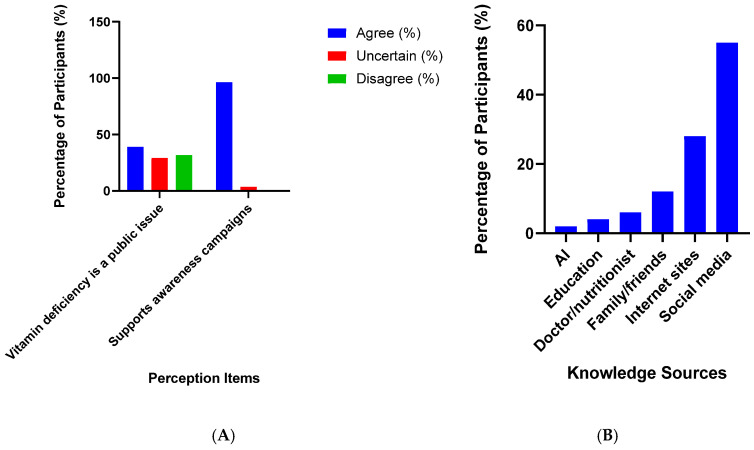
(**A**) Public perceptions and support for awareness campaigns addressing vitamin deficiency. Bars represent the proportion of participants who agreed, were uncertain, or disagreed with the statements. (**B**) Bar chart showing the percentage of participants who identified each source of information about vitamins and micronutrients.

Participants identified a range of sources for information about vitamins and micronutrients (Figure 4B). Social media was the most frequently cited source, mentioned by nearly 60% of respondents (Figure 4B). Other commonly reported sources included internet sites (approximately 35%) and family or friends (around 15%). Formal educational channels, such as doctors, nutritionists, or school education, were infrequently mentioned, each accounting for less than 10% of responses.

Regarding public perceptions, a substantial proportion of participants recognised vitamin deficiency as a significant public health issue, with 38.1% indicating agreement and a further 29.2% expressing uncertainty (Figure 4A). Only 31.7% did not perceive vitamin deficiency as a public concern. Support for awareness campaigns addressing vitamin deficiency was nearly universal, with 96.4% of respondents expressing support for such initiatives (Figure 4A).

## 4. Discussion

This study examined the sociodemographic characteristics, prevalence of micronutrient testing, and associated factors related to vitamin D, vitamin B12, and iron status among a large and diverse sample of 1652 individuals in Saudi Arabia. The findings reveal a population with a broad age distribution, a slight female majority, and high levels of education and marriage, reflecting key demographic trends in the region. The geographical diversity of the sample, with representation from major cities such as Makkah, Jeddah, and Riyadh, as well as smaller towns, enhances the generalisability of the results to the broader Saudi population.

One of the most striking results is the low prevalence of routine testing for key micronutrients. The majority of participants reported never having been tested for vitamin D (69.2%), vitamin B12 (68.9%), or iron (64.8%). This suggests that micronutrient screening is not a routine part of primary healthcare for many individuals in the Kingdom of Saudi Arabia. The low testing rate could be a result of a lack of awareness among the general population, or it could indicate that these tests are not routinely included in standard health checkups. Another explanation of low testing rate is that doctors may not inform patients about their test results. Thus, the patients are unaware whether these micronutrients have been tested or not. The low testing rates highlight a potential gap in proactive health management and could contribute to the underdiagnosis of deficiencies of vitamin D, vitamin B12, and iron.

The analysis revealed significant associations between vitamin D status and several sociodemographic, lifestyle, and health factors. For example, females had a higher proportion of vitamin D levels below the normal range compared to males. The significant association with marital status, where married participants had a higher proportion of normal results, could be related to lifestyle or dietary habits often associated with family life. The finding that individuals with chronic conditions, such as anaemia, osteoporosis, and high blood pressure, exhibit a higher frequency of below-normal vitamin D levels is medically important, but it needs further investigation to reveal a potential link between vitamin D deficiency and the pathogenesis of these chronic diseases. In addition, vitamin D is associated with several diseases in Saudi Arabia. For example, among patients with epilepsy (*n* = 524), the prevalence of low serum vitamin D levels was high (86.8%), and high vitamin D levels were associated with a 40% seizure reduction [35]. Another report showed that vitamin D deficiency was high among asthmatic children and adolescents [36]. Additionally, a study involving 2153 patients reported that vitamin D deficiency was diagnosed in 900 (41.8%) of them [37]. Despite the sunny weather, vitamin D deficiency remains prevalent in the Saudi population. In the Aseer Region, a study showed a high prevalence of vitamin D deficiency between children up to two years old [38]. Vitamin D deficiency was observed in 70.5% of the 484 children, with 45.9% having insufficient levels and 24.6% showing deficiency. Furthermore, researchers have indicated that iron and vitamin D deficiencies are still common as a malnutrition in the Saudi people [39]. These studies support the need for comprehensive micronutrient assessment, a goal addressed by our multi-nutrient analysis. However, our findings contrast with the encouraging trend reported in the central region of Saudi Arabia, which demonstrated a decline in Vitamin D deficiency prevalence across age groups from 2008 to 2017 [40]. This indicates the varied effectiveness of public health initiatives across different regions or populations and therefore, further investigation is needed to provide a comprehensive analysis of vitamin D deficiency status at the national level.

The results of vitamin B12 status revealed several noteworthy associations. While sociodemographic factors like age and gender did not show significant associations with deficiency, lifestyle and health factors did. Specifically, individuals who exercised more frequently and those without high cholesterol had a lower prevalence of vitamin B12 deficiency. This highlights the importance of an active lifestyle in maintaining overall health, which includes micronutrient balance. Our results could also suggest a possible link between vitamin B12 deficiency and certain chronic conditions. Individuals with anemia, high blood pressure, and diabetes were more likely to have below-normal vitamin B12 levels. This requires more clinical research to determine whether these conditions may either be a consequence of or contribute to vitamin B12 deficiency. Furthermore, this observed link between Vitamin B12 deficiency and diabetes is particularly relevant to existing studies in Saudi Arabia, for example, Vitamin B12 was significantly decreased in 366 patients with Type 2 Diabetes Mellitus (T2DM) receiving metformin [41]. This finding contrasts with another study that reported an increase in the serum of patients with diabetes mellitus who were using B12 as supplement therapy [42]. This emphasizes that vitamin B12 levels should be monitored in diabetic patients. Also, these clinical considerations are crucial for the therapeutic management of diabetes.

The network analysis shows that Vitamin B12 Test Result has strong associations with Takes Vitamin B12 Supplements and Vitamin D Test. Recent studies have investigated the awareness level and status of vitamin B12 in Saudi Arabia. For instance, a study showed that the level of awareness of vitamin B12 and its neurological consequences was moderate among 1314 respondents in Riyadh [43].

Iron deficiency was found to be most common in some age groups, a finding that warrants further investigation, as it may be linked to life-stage-specific factors such as diet, stress, or other health conditions. Unlike vitamin B12, iron status showed no significant association with gender or education level in this study. However, the connection between anemia and below-normal iron levels was exceptionally strong. This is an expected and clinically crucial finding, as iron deficiency is a leading cause of anaemia. The analysis also revealed significant associations between iron status and heart disease, high cholesterol, and high blood pressure, suggesting a complex interplay between iron metabolism and cardiovascular health. The network analysis corroborated these findings, highlighting the very strong link between Iron Test Result and Takes Iron Supplements. This reinforces the idea that supplementation is a strong indicator of an individual’s iron status and clinical history. The high prevalence of below-normal iron level in our study cohort is consistent with systemic reviews highlighting the significant burden of iron deficiency anemia in Saudi Arabis. The first systematic review showed that the occurrence of iron deficiency anemia in Saudi Arabia is exceptionally high, with some cities reporting rates as high as 67%, mainly impacting females, teenagers, children, and infants [44]. The second review and meta-analysis (2020–2024) reported that the prevalence of Iron Deficiency Anemia (IDA) among school-aged children was 26.8%, with fatigue, drowsiness, and concentration loss being the most common symptoms [45]. Anemia remains a significant health burden among Saudi children and adolescents. Additionally, research has shown that the prevalence of anemia in children and women varies between 12.5% and 70%, depending on the province [46]. In Makkah city, the prevalence of anemia was 57% in 461 respondents [47]. Furthermore, the same research indicated a statistically significant link between family history and the occurrence of anemia. In addition, a systematic review and meta-analysis reported a significant association between iron deficiency and obesity in children [48]. A comparative study showed that anemia and Body Mass Index (BMI) abnormalities can complicate asthma management [49]. Therefore, given the high prevalence and diverse clinical associations, routine screening and monitoring for iron deficiency across all age groups, especially in females, children, and adolescents, are critical for improving overall health outcomes.

The Cramér’s V analysis provided a clear picture of the variables most strongly associated with laboratory-confirmed micronutrient status. As expected, the strongest associations were between the tests themselves and their results (e.g., Iron Test and Iron Test Result). However, the substantial associations between micronutrient status and self-reported supplementation (Takes Iron Supplements and Takes Vitamin D Supplements) and clinical history (Anaemia with Iron Test Result) are of considerable clinical relevance. This finding could be leveraged for targeted screening and intervention. Conversely, sociodemographic factors like age, gender, and education level showed only minimal associations with actual test results, suggesting that while they may be linked to the decision to get tested, they are not the primary drivers of micronutrient deficiency itself. The network analysis further illuminated the complex relationships between different study variables. It visually confirms the strong connections between laboratory results and supplement use or chronic disease history, reinforcing the idea that health-related factors are more central to micronutrient status than demographic ones. The clustering of dietary and knowledge-based variables also suggests that individuals who are aware of health issues or have specific dietary habits tend to have other health-conscious behaviors.

Knowledge level regarding the symptom deficiency, sources and taking supplements of this micronutrient was investigated. Overall, our results indicate a moderate level of public awareness regarding micronutrient deficiencies, with knowledge of vitamin B12 being marginally higher than that of vitamin D and iron. However, the relatively low rates of supplement use highlight a gap between knowledge and preventive health behavior. For example, in this study, 42.4% of respondents knew the symptoms of vitamin D deficiency, whereas 42.7% were uncertain and 14.9% reported no knowledge. This finding of insufficient awareness is corroborated by another study from Saudi Arabia. Specifically, a study reported that only 17.4% of 466 participants recognized that exposure to sunlight is a primary source of vitamin D [50]. This research uncovers the insufficient awareness regarding vitamin D deficiency within the population of Al-Qunfudhah, Saudi Arabia. Our results and other studies’ findings indicate a variation in the health awareness level toward vitamin D in Saudi Arabia.

Similarly, in this study, 41.8% of respondents were aware of iron deficiency symptoms, while 28.6% were uncertain, and 29.5% reported no knowledge. Furthermore, the level of knowledge toward Iron deficiency Anemia (IDA) was studied among the Saudi population. A study reported that of the 385 participants, 42.5% exhibited strong knowledge of IDA, 48.1% possessed moderate knowledge, and 9.4% displayed inadequate knowledge in the Northern Border Region [51]. In Makkah, 84.50% of the 1395 participants demonstrated poor knowledge regarding iron deficiency anemia [52]. This significant variation underscores the local nature of the knowledge deficit shown in our study. Furthermore, while external research has demonstrated that educational programs can effectively enhance understanding and practices concerning IDA [53], our data on low supplement use suggest that a gap persists between improved knowledge and sustained positive health attitudes. Therefore, these findings collectively emphasize that more widespread, and consistent public health campaigns are required to address the prevalent lack of IDA awareness in Saudi Arabia.

Heath Knowledge sources and public perception were analysed regarding these micronutrients. The results highlight a critical reliance on informal sources of health information. Social media and the internet were the most frequently cited sources for information about vitamins and micronutrients, while formal channels like doctors and nutritionists were used far less. This trend underscores a potential challenge in ensuring that the public receives accurate, evidence-based health information. The high level of support for awareness campaigns (96.4%) and the widespread recognition of vitamin deficiency as a public health issue suggest a receptive audience for such initiatives. This presents a clear opportunity for public health authorities to launch targeted campaigns using popular media channels to disseminate accurate information and encourage routine testing.

## 5. Limitations and Future Directions

A key strength of this study is its large sample size and broad geographic representation, which increases the external validity of the findings. The use of a comprehensive survey instrument and advanced statistical techniques, such as Cramér’s V and network analysis, allowed for an exploration of the relationships between a wide range of variables. However, some limitations need to be addressed. This study employs a cross-sectional design, which relies on self-reported data, as participants’ memories and accuracy may be subject to recall bias. Future research could benefit from a longitudinal study design and the inclusion of clinical data to validate self-reported information and explore causal relationships. The questionnaire for collecting health data may not be ideal for uneducated people and participants older than 55 years, as we only received a small number from these groups. We suggest alternative methods such as analysing health records or conducting interviews with either the participants or their relatives.

The possible strong associations with chronic diseases and self-reported behaviors indicate a clear opportunity for targeted interventions. Public health campaigns should focus on increasing awareness of the symptoms of vitamin D, B12, and iron deficiency, encouraging at-risk populations to seek testing, and promoting a balanced diet. The future awareness campaigns could be particularly effective if they utilize social media platforms and other informal sources.

Future research should also develop efficient strategies to improve micronutrient screening and deficiency monitoring in Saudi Arabia.

## 6. Conclusions

Our study provides a comprehensive look at the status of vitamin D, vitamin B12, and iron within a diverse sample of the Saudi Arabian population. Our findings reveal a significant gap in proactive healthcare, evidenced by the alarmingly low rates of routine micronutrient testing. This suggests that deficiencies may be widespread but largely undiagnosed, which could have serious public health consequences. Furthermore, while 38.1% recognize micronutrient deficiency as a public health concern and 96% of participants support awareness campaigns, these attitudes do not translate into testing-seeking behavior. Our findings show several critical associations that have significant clinical and public health implications. We found that micronutrient status is possibly linked to chronic diseases such as anaemia, diabetes and high blood pressure. This highlights a clear opportunity for targeted interventions. The healthcare providers should consider routine screening for these micronutrients, particularly in patients with these chronic conditions. For example, healthcare providers should implement routine vitamin B12 screening in diabetic patients (particularly those on metformin), individuals with anaemia, or hypertension; perform vitamin D assessment in patients with chronic diseases; and conduct iron evaluation in specific age groups or those with anaemia or cardiovascular disease. In addition, the strong association between supplementation and lab-confirmed status validates the use of self-reported data as a valuable indicator of an individual’s health history.

These findings collectively underscore that closing the micronutrient testing gap in Saudi healthcare requires integrated approaches that address both healthcare system capacity and sustained public health engagement.

The widespread reliance on social media for health information, coupled with a strong public perception of awareness campaigns, presents a unique opportunity for health authorities. Future public health initiatives should effectively utilize these platforms to disseminate accurate, evidence-based information. Health campaigns should focus on increasing public awareness of deficiency symptoms and encouraging at-risk populations to seek routine testing and consult with a healthcare professional.

Future research should also determine the causal relationships between micronutrient deficiencies and some chronic diseases to better inform clinical guidelines and public health policies.

## Figures and Tables

**Table 1:** Distribution of Sociodemographic Characteristics Among Study Participants. This table summarises the age, gender, marital status, educational attainment, and city of residence for all respondents (*n* = 1652).

Characteristics		*n*	%
**Age**	18–25	213	12.9
	25–35	359	21.7
	35–45	535	32.4
	45–55	414	25.1
	55+	131	7.9
**Gender**	Female	869	52.6
	Male	783	47.4
**Marital Status**	Married	1359	82.3
	Other	82	5.0
	Single	211	12.8
**Education Level**	High School	168	10.2
	Less than High School	2	0.1
	Postgraduate	265	16.0
	University	1217	73.7
**Saudi Provinces**	Makkah Province	356	21.5
	Eastern Province	327	19.8
	Al-Jawf Province	200	12.1
	Asir Province	159	9.6
	Al-Qassim Province	118	7.1
	Tabuk Province	94	5.7
	Riyadh Province	90	5.4
	Al-Madinah Province	68	4.1
	Jazan Province	64	3.9
	Hail Province	62	3.8
	Najran Province	54	3.3
	Northern Borders Province	49	3.0
	Al-Bahah Province	11	0.7

**Table 2:** Self-reported of Laboratory Testing and Deficiency for Vitamin D, Vitamin B12, and Iron.

Self-Reported of Laboratory Testing		*n*	%
**Vitamin D Test** **Result**	Below Normal	126	7.6%
	Normal	383	23.2%
	Not Tested	1143	69.2%
**Vitamin B12** **Test Result**	Below Normal	91	5.5%
	Normal	423	25.6%
	Not Tested	1138	68.9%
**Iron Test** **Result**	Below Normal	116	7.0%
	Don’t Know	1	0.1%
	Normal	464	28.1%
	Not Tested	1071	64.8%

**Table 3:** (**a**) Association of Vitamin D Status with Sociodemographic and Clinical Characteristics. The table summarises the frequencies and proportions of participants with below-normal, normal, and untested Vitamin D results, by demographic, lifestyle, and clinical factors. Chi-squared statistics (χ^2^) and *p*-values are shown for each comparison. (**b**) Association of Vitamin B12 Status with Sociodemographic and Clinical Characteristics. The table summarises the frequencies and proportions of participants with below-normal, normal, and untested Vitamin B12 results, by demographic, lifestyle, and clinical factors. Chi-squared statistics (χ^2^) and *p*-values are shown for each comparison. (**c**) Association of Iron Status with Sociodemographic and Clinical Characteristics. The table summarises the frequencies and proportions of participants with below-normal, normal, and untested Iron results, by demographic, lifestyle, and clinical factors. Chi-squared statistics (χ^2^) and *p*-values are shown for each comparison. Values shown in bold indicate statistically significant associations (*p* < 0.05).

**(a)**											
**Characteristics**				**Vitamin D Status**							
				**Below Normal**		**Normal**		**Not Tested**		***χ*** **^2^**	***p*** **-Value**
				***n***	**%**	***n***	**%**	***n***	**%**		
**Age**		18–25		21	1.3	37	2.2	155	9.4	21.166	**0.020**
		25–35		30	1.8	99	6.0	230	13.9		
		35–45		47	2.8	111	6.7	377	22.8		
		45–55		17	1.0	101	6.1	296	17.9		
		55+		11	0.7	35	2.1	85	5.1		
**Gender**		Female		71	4.3	177	10.7	621	37.6	8.348	**0.015**
		Male		55	3.3	206	12.5	522	31.6		
**Marital Status**		Married		79	4.8	329	19.9	951	57.6	43.639	**<0.001**
		Other		8	0.5	17	1.0	57	3.5		
		Single		39	2.4	37	2.2	135	8.2		
**Education Level**		High School		17	1.0	47	2.8	104	6.3	16.290	**0.012**
		<High School		1	0.1	0	0.0	1	0.1		
		Postgraduate		25	1.5	71	4.3	169	10.2		
		University		83	5.0	265	16.0	869	52.6		
**Exercise Frequency**		3–5 days/week		43	2.6	163	9.9	378	22.9	44.371	**<0.001**
		Daily		7	0.4	38	2.3	78	4.7		
		I do not exercise		34	2.1	40	2.4	140	8.5		
		<3 days/week		42	2.5	142	8.6	547	33.1		
**Smoking Status**		No		110	6.7	326	19.7	1035	62.7	9.106	**0.011**
		Yes		16	1.0	57	3.5	108	6.5		
**Primary Food Source**		Home-cooked food		43	2.6	121	7.3	276	16.7	13.687	**0.008**
		Mix of both		75	4.5	243	14.7	814	49.3		
		Restaurants/fast food		8	0.5	19	1.2	53	3.2		
**Meat Diet**		No		28	1.7	105	6.4	359	21.7	5.915	0.052
		Yes		98	5.9	278	16.8	784	47.5		
**Vegetarian Diet**		No		113	6.8	365	22.1	1110	67.2	17.738	**<0.001**
		Yes		13	0.8	18	1.1	33	2.0		
**High Protein Diet**		No		91	5.5	264	16.0	763	46.2	1.910	0.385
		Yes		35	2.1	119	7.2	380	23.0		
**Fast Food Diet**		No		96	5.8	311	18.8	937	56.7	2.513	0.285
		Yes		30	1.8	72	4.4	206	12.5		
**Diabetes**		No		115	7.0	351	21.2	1081	65.4	5.432	0.066
		Yes		11	0.7	32	1.9	62	3.8		
**Anemia**		No		106	6.4	373	22.6	1103	66.8	46.071	**<0.001**
		Yes		20	1.2	10	0.6	40	2.4		
**Osteoporosis**		No		122	7.4	380	23.0	1141	69.1	19.370	**<0.001**
		Yes		4	0.2	3	0.2	2	0.1		
**Heart Disease**		No		118	7.1	365	22.1	1109	67.1	5.317	0.070
		Yes		8	0.5	18	1.1	34	2.1		
**High Cholesterol**		No		111	6.7	369	22.3	1129	68.3	53.239	**<0.001**
		Yes		15	0.9	14	0.8	14	0.8		
**Other Diseases**		No		110	6.7	365	22.1	1061	64.2	9.423	**0.009**
		Yes		16	1.0	18	1.1	82	5.0		
**High Blood Pressure**		No		108	6.5	352	21.3	1089	65.9	20.695	**<0.001**
		Yes		18	1.1	31	1.9	54	3.3		
**(b)**											
**Characteristics**				**Vitamin B12 Status**							
				**Below Normal**		**Normal**		**Not Tested**		***χ*** **^2^**	***p*** **-Value**
				***n***	**%**	***n***	**%**	***n***	**%**		
**Age**		18–25		9	0.5	43	2.6	161	9.7	11.676	0.307
		25–35		14	0.8	98	5.9	247	15.0		
		35–45		36	2.2	133	8.1	366	22.2		
		45–55		21	1.3	114	6.9	279	16.9		
		55+		11	0.7	35	2.1	85	5.1		
**Gender**		Female		53	3.2	221	13.4	595	36.0	1.228	0.541
		Male		38	2.3	202	12.2	543	32.9		
**Marital Status**		Married		71	4.3	358	21.7	930	56.3	3.747	0.441
		Other		7	0.4	19	1.2	56	3.4		
		Single		13	0.8	46	2.8	152	9.2		
**Education Level**		High School		14	0.8	45	2.7	109	6.6	7.654	0.265
		<High School		0	0.0	0	0.0	2	0.1		
		Postgraduate		15	0.9	79	4.8	171	10.4		
		University		62	3.8	299	18.1	856	51.8		
**Exercise Frequency**		3–5 days/week		38	2.3	173	10.5	373	22.6	24.119	<0.001
		Daily		7	0.4	31	1.9	85	5.1		
		I do not exercise		21	1.3	48	2.9	145	8.8		
		<3 days/week		25	1.5	171	10.4	535	32.4		
**Smoking Status**		No		82	5.0	363	22.0	1026	62.1	6.073	0.048
		Yes		9	0.5	60	3.6	112	6.8		
**Primary Food Source**		Home-cooked food		29	1.8	123	7.4	288	17.4	3.959	0.412
		Mix of both		59	3.6	279	16.9	794	48.1		
		Restaurants/fast food		3	0.2	21	1.3	56	3.4		
**Meat Diet**		No		29	1.8	127	7.7	336	20.3	0.237	0.888
		Yes		62	3.8	296	17.9	802	48.5		
**Vegetarian Diet**		No		87	5.3	404	24.5	1097	66.4	0.725	0.696
		Yes		4	0.2	19	1.2	41	2.5		
**High Protein Diet**		No		50	3.0	282	17.1	786	47.6	7.948	0.019
		Yes		41	2.5	141	8.5	352	21.3		
**Fast Food Diet**		No		70	4.2	339	20.5	935	56.6	2.077	0.354
		Yes		21	1.3	84	5.1	203	12.3		
**Diabetes**		No		79	4.8	403	24.4	1065	64.5	9.024	0.011
		Yes		12	0.7	20	1.2	73	4.4		
**Anemia**		No		81	4.9	404	24.5	1097	66.4	11.420	0.003
		Yes		10	0.6	19	1.2	41	2.5		
**Osteoporosis**		No		90	5.4	420	25.4	1133	68.6	0.960	0.619
		Yes		1	0.1	3	0.2	5	0.3		
**Heart Disease**		No		88	5.3	405	24.5	1099	66.5	0.635	0.728
		Yes		3	0.2	18	1.1	39	2.4		
**High Cholesterol**		No		85	5.1	406	24.6	1118	67.7	12.270	0.002
		Yes		6	0.4	17	1.0	20	1.2		
**Other Diseases**		No		82	5.0	392	23.7	1062	64.3	1.413	0.493
		Yes		9	0.5	31	1.9	76	4.6		
**High Blood Pressure**		No		82	5.0	385	23.3	1082	65.5	10.907	0.004
		Yes		9	0.5	38	2.3	56	3.4		
**(c)**											
**Characteristics**		**Iron Status**									
		**Below Normal**		**Don’t Know**		**Normal**		**Not Tested**		***χ*** **^2^**	***p*** **-Value**
		***n***	**%**	***n***	**%**	***n***	**%**	***n***	**%**		
**Age**	18–25	21	1.3	0	0.0	44	2.7	148	9.0	16.133	0.373
	25–35	22	1.3	0	0.0	100	6.1	237	14.3		
	35–45	41	2.5	1	0.1	163	9.9	330	20.0		
	45–55	22	1.3	0	0.0	119	7.2	273	16.5		
	55+	10	0.6	0	0.0	38	2.3	83	5.1		
**Gender**	Female	67	4.1	0	0.0	263	15.9	539	32.6	7.667	0.053
	Male	49	3.0	1	0.1	201	12.2	532	32.2		
**Marital Status**	Married	86	5.2	0	0.0	388	23.5	885	53.6	14.280	**0.027**
	Other	6	0.4	0	0.0	22	1.3	54	3.3		
	Single	24	1.5	1	0.1	54	3.3	132	8.0		
**Education Level**	High School	17	1.0	0	0.0	47	2.8	104	6.3	13.831	0.128
	<High School	0	0.0	0	0.0	1	0.1	1	0.1		
	Postgraduate	18	1.1	1	0.1	89	5.4	157	9.5		
	University	81	4.9	0	0.0	327	19.8	809	49.0		
**Exercise Frequency**	3–5 days/week	36	2.2	1	0.1	187	11.3	360	21.8	14.601	**0.102**
	Daily	8	0.5	0	0.0	36	2.2	79	4.8		
	I do not exercise	22	1.3	0	0.0	60	3.6	132	8.0		
	<3 days/week	50	3.0	0	0.0	181	11.0	500	30.3		
**Smoking Status**	No	105	6.4	1	0.1	412	24.9	953	57.7	0.415	0.937
	Yes	11	0.7	0	0.0	52	3.1	118	7.1		
**Primary Food Source**	Home-cooked food	34	2.1	0	0.0	137	8.3	269	16.3	10.461	0.107
	Mix of both	76	4.6	1	0.1	296	17.9	759	45.9		
	Restaurants/fast food	6	0.4	0	0.0	31	1.9	43	2.6		
**Meat Diet**	No	31	1.9	0	0.0	144	8.7	317	19.2	1.308	0.727
	Yes	85	5.1	1	0.1	320	19.4	754	45.6		
**Vegetarian Diet**	No	110	6.7	1	0.1	437	26.5	1040	63.0	8.038	0.045
	Yes	6	0.4	0	0.0	27	1.6	31	1.9		
**High Protein Diet**	No	78	4.7	1	0.1	307	18.6	732	44.3	1.193	0.755
	Yes	38	2.3	0	0.0	157	9.5	339	20.5		
**Fast Food Diet**	No	89	5.4	1	0.1	371	22.5	883	53.5	3.308	0.347
	Yes	27	1.6	0	0.0	93	5.6	188	11.4		
**Diabetes**	No	108	6.5	1	0.1	437	26.5	1001	60.6	0.408	0.939
	Yes	8	0.5	0	0.0	27	1.6	70	4.2		
**Anemia**	No	82	5.0	1	0.1	448	27.1	1051	63.6	195.296	**<0.001**
	Yes	34	2.1	0	0.0	16	1.0	20	1.2		
**Osteoporosis**	No	115	7.0	1	0.1	460	27.8	1067	64.6	1.663	0.645
	Yes	1	0.1	0	0.0	4	0.2	4	0.2		
**Heart Disease**	No	108	6.5	0	0.0	448	27.1	1036	62.7	30.516	**<0.001**
	Yes	8	0.5	1	0.1	16	1.0	35	2.1		
**High Cholesterol**	No	112	6.8	0	0.0	444	26.9	1053	63.7	46.675	**<0.001**
	Yes	4	0.2	1	0.1	20	1.2	18	1.1		
**Other Diseases**	No	107	6.5	1	0.1	425	25.7	1003	60.7	2.274	0.517
	Yes	9	0.5	0	0.0	39	2.4	68	4.1		
**High Blood Pressure**	No	107	6.5	0	0.0	437	26.5	1005	60.8	15.646	**0.001**
	Yes	9	0.5	1	0.1	27	1.6	66	4.0		

**Table 4:** Knowledge Level of Deficiency Symptoms, Sources, and Supplementation of micronutrients.

**Q**	** Do You Know the Symptoms of Deficiency? **					
	**Yes**		**May Be**		**No**	
	***n***	**%**	***n***	**%**	***n***	**%**
** Vitamin D **	700	42.4	706	42.7	246	14.9
** Vitamin B12 **	765	46.3	362	21.9	525	31.8
**Iron**	691	41.8	473	28.6	488	29.5
**Q**	** Do You Know the Food Sources of These Micronutrients? **					
	**Yes**		**May Be**		**No**	
	***n***	**%**	**N**	**%**	***n***	**%**
** Vitamin D **	719	43.5	533	32.3	400	24.2
** Vitamin B12 **	744	45	307	18.6	601	36.4
**Iron**	863	52.2	331	20	458	27.7
**Q**	** Do You Take Supplements? **					
	** Yes **			** No **		
	***n***	**%**		***n***		**%**
** Vitamin D **	331	20		1321		80
** Vitamin B12 **	299	18.1		1353		81.9
**Iron**	371	22.5		1281		77.5

## Data Availability

The original contributions presented in this study are included in the article/Supplementary Materials. Further inquiries can be directed to the corresponding author.

## Supplementary Materials

The following supporting information can be downloaded at: https://www.mdpi.com/article/10.3390/nu17243897/s1, File S1: Questionnaire; Table S1. Participants were geographically diverse, representing a wide range of cities across the Kingdom of Saudi Arabia. The most significant proportion of respondents resided in Makkah (11.0%) and Jeddah (6.6%), followed by Dammam (5.1%), Al Jowf (5.1%), Riyadh (5.1%), and Khafji (4.1%). Other notable cities included Tabuk (4.1%), Hail (3.8%), Jazan (3.9%), Aseer (3.6%), and Tubarjal (3.5%). Several cities contributed smaller proportions to the sample, reflecting a broad national distributiom; Figure S1. Exercise frequency: 7.4% daily, 44.2% less than 3 times in a week, 35.4% 3–5 days in a week and 13% don’t exercise; Figure S2. Diet types: 3.9% vegetarian diet, 33.3% high protein diet, 70.2% mix of both diets and 24.3% fast food diet; Figure S3. Time of sunlight exposure: 41.6% less than 10 mins, 54.2% more than 30 mins and 4.1% don’t exposure at all.
