# Supplementary material for: Micronutrient Testing, Supplement Use, and Knowledge Gaps in a National Adult Population: Evidence from Saudi Arabia
Source: Nutrients. 2025 Dec 12;17(24):3897. doi: 10.3390/nu17243897 (PMC12736049; doi:10.3390/nu17243897)
Supplement: Supplementary file 1 [file nutrients-17-03897-s001.zip › nutrients-3995700-supplementary.pdf]

Supplementary Materials file S1：Questionnaire

Informed Consent Form for Research Participation:

Study Title: Micronutrient Testing, Supplement Use, and Knowledge Gaps in a National Adult Population: Evidence from Saudi Arabia

Principal Investigator: Dr. Abdulmajeed Fahad Alrefaei, Biology Department, Umm Al-Qura University. afrefaei@uqu.edu.sa

Co-Investigator(s): Dr. Saeed M Kabrah, Department of Clinical Laboratory Sciences, Faculty of Applied Medical Sciences, Umm Al-Qura University

Ethical Approval Reference No.: HAPO-02-K-012-2025-03-2621

1. Introduction

You are being invited to take part in a research study conducted by researchers from Umm Al-Qura University. Before you agree to participate, please read the following information carefully and ask any questions you may have.

Participation in this study is voluntary. You may choose not to participate or to withdraw at any time without any penalty or loss of benefits.

2. Background and Purpose of the Study

Micronutrient deficiencies, especially of vitamin D, vitamin B12, and iron, are common worldwide and can have significant health impacts. In Saudi Arabia, these deficiencies are increasingly recognised as a public health concern.

The aims of this study are:

*Determine the prevalence of laboratory-confirmed deficiency and testing for vitamin D,

vitamin B12, and iron among adults in Saudi Arabia.

*Identify sociodemographic, lifestyle, and clinical factors associated with these deficiencies.

*Assess public knowledge, attitudes, and awareness regarding micronutrient status through this questionnaire.

3. Confidentiality

All information collected in this study will be kept strictly confidential. Your data will be coded, and your information will not appear in any publication or presentation. Only the research team will have access to the data.

4. Voluntary Participation and Right to Withdraw

Your participation is entirely voluntary. You may refuse to participate or withdraw at any time without any negative consequences.

5. Participant Statement and Consent

I have read and understood the information provided above, and I voluntarily agree to participate in this study:

	Yes
	NO

*If you have any concerns about your rights as a participant, you may contact the Principal Investigator via this email; afrefaei@uqu.edu.sa

Section 1: Personal Information

Age:

	18-25
	25-35
	35-45
	45-55
	55-65
	More than 65

Gender:

	Male
	Female

Marital Status:

	Single
	Married
	other

Educational level:

	Less than High School
	Secondary School
	Undergraduate
	Postgraduate

Your City:

Section 2: Health habits:

How often do you practice sports/exercise?

	Daily
	3-5 days each week
	3 times weekly
	No exercise

Do you smoke?

	Yes
	No

What is your main source of food?

	Home-cooked food

	Restaurants or fast food"
	A mix of both

What is your dietary pattern? (You can choose more than one option)

	Vegetarian
	A mix of meat and vegetables
	High-protein
	Relies on fast food

Section 3: Health Data:

Do you currently or have you previously suffered from any of the following conditions? (You can choose more than one option)

	High blood pressure
	Diabetes
	Anemia
	High cholesterol
	Osteoporosis
	Cardiovascular diseases
	Other diseases
	No diseases

Section vitamin D:

Have you ever had your Vitamin D levels checked?

	Yes
	No

If the answer is 'Yes', what was the result of the last test?

	Normal
	Below normal
	I have not had the test

How often are you exposed to sunlight daily?

	Less than 10 minutes
	More than 30 minutes
	I am not exposed to the sun

Do you know the symptoms of Vitamin D deficiency?

	Yes
	No

	May Be

Do you have knowledge of the dietary sources of Vitamin D?

	Yes
	No
	May Be

Do you take Vitamin D supplements?

	Yes
	No

Section vitamin B12:

Have you ever had your Vitamin B_{12} levels checked?

	Yes
	No

If the answer is 'Yes', what was the result of the last test?

	Normal
	Below normal
	I have not had the test

Do you know the symptoms of Vitamin B_{12} deficiency?

	Yes
	No
	May Be

Do you have knowledge of the dietary sources of Vitamin B_{12}?

	Yes
	No
	May Be

Do you take Vitamin B_{12} supplements?

	Yes
	No

Section Iron:

Have you ever had your Iron levels checked?

	Yes
	No

If the answer is 'Yes', what was the result of the last test?

	Normal
	Below normal
	I have not had the test

Do you know the symptoms of Iron deficiency?

	Yes
	No
	May Be

Do you have knowledge of the dietary sources of Iron?

	Yes
	No
	May Be

Do you take Iron supplements?

	Yes
	No

Section Health Awareness Assessment:

What is the source of your knowledge about vitamins and minerals?

	Doctor/Nutritionist
	The Media
	Internet websites
	Family/Friends
	Social media
	others

Do you think that vitamin and mineral deficiency is a widespread health problem in your community?

	Yes
	No
	May Be

Do you support launching awareness campaigns to improve nutritional and health awareness in your community?

	Yes
	No

Thanks and Appreciation:

Thank you for your time and contribution to this scientific survey.

Table S1. shows that Participants were geographically diverse, representing a wide range of cities across the Kingdom of Saudi Arabia. The most significant proportion of respondents resided in Makkah (11.0%) and Jeddah (6.6%), followed by Dammam (5.1%), Al Jowf (5.1%), Riyadh (5.1%), and Khafji (4.1%). Other notable cities included Tabuk (4.1%), Hail (3.8%), Jazan (3.9%), Aseer (3.6%), and Tubarjal (3.5%). Several cities contributed smaller proportions to the sample, reflecting a broad national distribution.

City	Abha	33	2.0%
	Al Bahah	12	0.7%
	Al Hofuf	52	3.1%
	Al Jamom	8	0.5%
	Al Jowf	84	5.1%
	Al Jubail	30	1.8%
	Al Khobar	42	2.5%
	Al Qassim	40	2.4%
	Al Qatif	2	0.1%
	Al Qurayyat	31	1.9%
	Al Uferiah	8	0.5%
	Arar	29	1.8%
	Aseer	60	3.6%
	Bisha	39	2.4%
	Buraydah	31	1.9%
	Damam	85	5.1%
	Dhahran	45	2.7%
	Duba	27	1.6%
	Hail	62	3.8%
	Jazan	64	3.9%
	Jeddah	109	6.6%
	Khafji	68	4.1%
	Khamis Mushait	27	1.6%
	Madinah	37	2.2%
	Makkah	181	11.0%
	Najran	54	3.3%

	Ranyah	2	0.1%
	Riyadh	84	5.1%
	Sakaka	28	1.7%
	Tabuk	67	4.1%
	Taif	56	3.4%
	Tubarjal	57	3.5%
	Turaif	20	1.2%
	Unaizah	47	2.8%
	Yanbu	31	1.9%

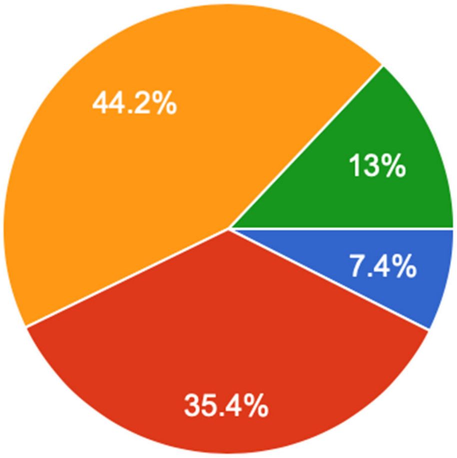

Figure S 1. Exercise frequency: 7.4% daily, 44.2% less than 3 times in a week, 35.4% 3-5 days in a week and 13% don't exercise.

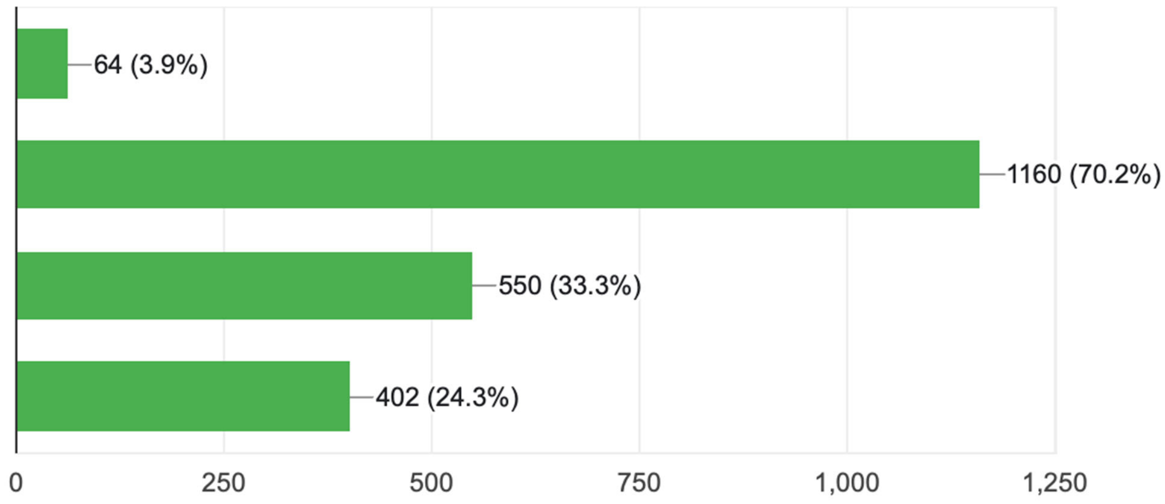

Figure S 2. Diet types: 3.9% vegetarian diet, 33.3% high protein diet, 70.2% mix of both diets and 24.3% fast food diet.

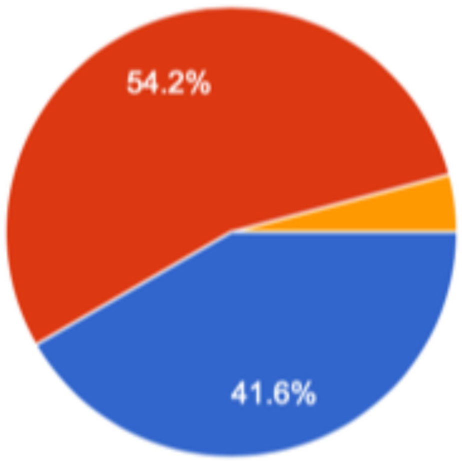

Figure S 3. Time of sunlight exposure: 41.6 % less than 10 mins, 54.2% more than 30 mins and 4.1% don't exposure at all.
